# On the Structural Context and Identification of Enzyme Catalytic Residues

**DOI:** 10.1155/2013/802945

**Published:** 2013-02-03

**Authors:** Yu-Tung Chien, Shao-Wei Huang

**Affiliations:** Department of Medical Informatics, Tzu Chi University, 701 Zhongyang Road, Section 3, Hualien 97004, Taiwan

## Abstract

Enzymes play important roles in most of the biological processes. Although only a small fraction of residues are directly involved in catalytic reactions, these catalytic residues are the most crucial parts in enzymes. The study of the fundamental and unique features of catalytic residues benefits the understanding of enzyme functions and catalytic mechanisms. In this work, we analyze the structural context of catalytic residues based on theoretical and experimental structure flexibility. The results show that catalytic residues have distinct structural features and context. Their neighboring residues, whether sequence or structure neighbors within specific range, are usually structurally more rigid than those of noncatalytic residues. The structural context feature is combined with support vector machine to identify catalytic residues from enzyme structure. The prediction results are better or comparable to those of recent structure-based prediction methods.

## 1. Introduction

Understanding the molecular mechanisms of enzyme catalysis is important in studies of various complicated biological processes. The number of protein structures deposited to the Protein Data Bank [1] has increased rapidly in the past decade. However, the function and catalytic residues of a large fraction of enzymes are not well studied and understood. Experimental methods which are used to identify enzyme catalytic residues, like site-directed mutagenesis, are time consuming and expensive. Computational methods designed to identify catalytic residues are needed to efficiently handle the huge number of proteins whose catalytic sites are not determined.

Many methods have been developed to predict protein catalytic sites based on information extracted from protein seqence and structure. One of the most direct strategies is based on finding homologous enzymes whose function and catalytic residues are already known [2–6]. Catalytic residues of a novel protein are identified by using sequence or structure similarity search with enzymes whose catalytic residues were well annotated. However, there are still limitations for such methods based on homology search. First, homologous enzymes whose function and catalytic sites are already known are needed. Second, proteins of similar tertiary structures do not always have completely identical function [7]. There are also examples showing that proteins of different tertiary structures have the same function [8]. 

To directly identify catalytic sites from single protein structure without needing homology information, it is important to study the fundamental differences between catalytic residues and noncatalytic residues. Sacquin-Mora et al. [9] used the computation of a force constant, that is, the ease of moving a given residue with respect to the other residues in the protein, to identify catalytic residues and found that the catalytic residues usually have higher force constant. Ben-Shimon and Eisenstein [10] found that the catalytic residues are often located near the small fractions of the exposed residues closest to the center of the protein. Amitai et al. [11] converted protein to a network in which the residues are vertices and their interactions are edges and showed that the central hubs in the network are usually functional important residues or residues having direct contact with them. Wie et al. [12] developed a method, Theoretical Microscopic Titration Curves (THEMATICS), which computes residue electrostatic properties from protein structure, to identify catalytic residues. The THEMATICS method was then combined with geometry features derived from protein structure to predict catalytic residues from enzyme structure using a monotonicity-constrained maximum likelihood approach, called Partial Order Optimum Likelihood (POOL) [13]. A more recent method, EXIA [14], successfully identifies catalytic residues based on residue side chain orientation of single enzyme structure without needing structure or sequence homology information.

In this study, we first analyzed the structural context of catalytic and noncatalytic residues based on their sequence and structure neighbors. We show that catalytic residues are usually located in structurally more rigid environment than noncatalytic residues. The sequence or structural neighboring residues within specific range of catalytic residues have distinct structural features. We further combined the structural context features and support vector machine to identify catalytic residues from protein structure.

## 2. Methods

### 2.1. Calculation of Structural Context

The weighted-contact number model (WCN) [15, 16] is used to calculate structural flexibility of residue environment. WCN is highly correlated to experimental B-factor and order parameter of protein structure solved by nuclear magnetic resonance. The WCN of the *i*th residue is based on the distances between the *i*th residue and all the other residues in the enzyme, as in

(1) $$\begin{array}{c} D_{i}=\sum_{j\neq i}^{N}\frac{1}{r_{ij}^{2}}, \end{array}$$

where *N* is total number of residues in the enzyme, and *r*_*ij*_ is the distance between *i*th and*j*th residues. The coordinate of C*α* atom is used to represent the position of the residue.

There are two types of structural context: sequence neighbor flexibility (SEQ) and structure neighbor flexibility (STR). The SEQ of the *i*th residue is defined as the average structural flexibility of the *i*th residue and its flanking residues on sequence as in

(2) $$\begin{array}{c} {\text{SEQ}}_{i}=\frac{\sum_{x\;=\;i-n}^{x\;\leq \;i+n}D_{x}^{-1}}{\left(2n+1\right)}, \end{array}$$

where residues *i* − *n* to *i* + *n* are the nearest *n* neighbors of the *i*th residue on sequence. WCN is inverted for an easy comparison with B-factor. If *x* is out of the range of the sequence, it is simply ignored in the calculation. The STR of the *i*th residue is defined as the average structural flexibility of the *i*th residue and residues whose distance to the *i*th residue are smaller than a cut-off value as in

(3) $$\begin{array}{c} {\text{STR}}_{i}=\frac{\sum_{x\in M}D_{x}^{-1}}{m}, \end{array}$$

where *M* is a subset of residues whose distance to the *i*th residue is smaller than the cut-off distance and *m* is the number of residues in the subset. The concept of SEQ and STR is extended from our previous work [17], which only considered the nearest two sequence neighbors.

### 2.2. Normalization of Structural Context and B-Factor Profiles

The SEQ, STR, and B-factor are normalized to their corresponding *z*-scores:

(4) $$\begin{array}{c} z_{x}=\frac{x-\overset{-}{x}}{\sigma_{x}}, \end{array}$$

where $\overset{-}{x}$ and *σ*_*x*_ are the mean and standard deviations of *x* of a given protein. In this work, *x* is SEQ, STR, or B-factor from X-ray crystallographic structures. For a given protein, the mean and standard deviations are calculated based on the scores of the protein. The scores of the protein are then normalized according to its mean and standard deviations. The normalized SEQ, STR, and B-factor are referred to as *Z*_SEQ_, *Z*_STR_, and *Z*_B_, respectively. For convenience, the normalized SEQ, STR, and normalized B-factor profiles are simply called SEQ profile, STR profile and B-factor profile.

### 2.3. The Support Vector Machine

SVM finds the separating hyperplane with the largest distance between two classes. However, the data being classified may not always be linearly separable in the space. It was proposed that the original space be mapped into a higher-dimensional space, making the separation easier in that space. The support vector machine method (SVM) has been widely applied to many bioinformatics studies: protein-fold assignment [18, 19], subcellular localization prediction [20, 21], secondary-structure prediction [22–24], and other biological pattern-classification problems [25–28]. SVMs perform well in these classification problems when compared to other machine-learning methods because of thier convenient classifier's capacity control and avoidance of overfitting. In this work, the software package LIBSVM [29] version 3.11 was used.

Here we used SVM to predict catalytic residues using the structural context features, SEQ and STR, as input features. The feature vector for a residue is one of these features or their combinations: *Z*_SEQ_, *Z*_STR_, *Z*_B_, or binary coding of amino acid type. The common problem encountered in enzyme catalytic site prediction using SVM is the extremely unbalanced ratio of catalytic residues and noncatalytic residues. A well-used strategy is to randomly select subsets which have a balanced ratio between catalytic and noncatalytic residues when training [30]. Here, a 5-fold cross-validation procedure was used for performance measurement. For each fold, the training data was a randomly selected balanced subset of residues by subsampling noncatalytic residues. The *cost* and *gamma* are parameters used in model training and kernel function of LIBSVM and need to be to tuned for optimal prediction results. These parameters are tuned independently using 5-fold cross-validation for each training dataset. In addition to cost and gamma parameters, other settings used in the SVM include the type of SVM: C-SVC; the type of kernel function: radial basis function. Other parameters not mentioned here are set as their default value in the LIBSVM software.

### 2.4. Sequence Conservation Score

For comparison with the POOL method, sequence conservation which includes evolutionary information is used as training feature in some predictions reported here. Sequence conservation is from position-specific substitution matrix (PSSM) generated by PSI-Blast [31] for each protein. PSI-Blast is set to search against the nonredundant (nr) database for three iterations with default *E*-value threshold of 5 × 10^−3^. The sequence conservation score is directly taken from the “information per position” column in the PSSM profile.

### 2.5. Dataset

The dataset used in this work is collected from Catalytic Site Atlas (CSA) [32] version 2.2.10 using BlastClust [31]. The dataset contains 760 proteins with pairwise sequence identity ≤30%, including a total of 592,382 residues in which 2,355 residues are catalytic sites. All heteroatoms, ligands, and nonprotein molecules are removed. The dataset is referred to as E760 dataset. 

### 2.6. Evaluation of Prediction Performance

Sensitivity, specificity, and Matthew's correlation coefficient (MCC) were used for performance measure as follows:

(5) $$\begin{array}{c} \text{Sensitivity}=\frac{\text{TP}}{\left(\text{TP}+\text{FN}\right)},\\ \text{Specificity}=\frac{\text{TN}}{\left(\text{TN}+\text{FP}\right)},\\ \text{MCC}=\frac{\text{TP}\times \text{TN}-\text{FP}\times \text{FN}}{\sqrt{\left(\text{TP}+\text{FP}\right)\times \left(\text{TP}+\text{FN}\right)\times \left(\text{TN}+\text{FN}\right)\times \left(\text{TN}+\text{FP}\right)}}, \end{array}$$

where TP, FP, TN, and FN are the number of true positive, false positive, true negative, and false negative, respectively. A catalytic residue is either TP when correctly predicted to be catalytic residue or FP when incorrectly predicted to be noncatalytic residue. A noncatalytic residue is either TN when correctly predicted to be noncatalytic residue or FN when incorrectly predicted to be catalytic residue. We used MCC to evaluate the performances because MCC takes into account true and false positives and negatives and is a balanced measure especially when the numbers of positives (catalytic residues) and negatives (noncatalytic residues) are extremely unbalanced. Note that the MCC, sensitivity, and specificity reported here are based on balanced data, that is, the numbers of catalytic and noncatalytic residues are equal. They were only used to compare the results between different features in this paper but not used to compare with other methods. The Receiver Operating Characteristic (ROC) curve was calculated based on unbalanced data and was used to compare prediction results with other methods. The ROC curve was plotted by averaging per-protein ROC curve as used in [13].

## 3. Results and Discussions

First, we discuss the distributions of *Z*_SEQ_, *Z*_STR_, and *Z*_B_ of catalytic residues and noncatalytic residues for the E760 dataset. Then we show the prediction results based on *Z*_SEQ_ profile, *Z*_B_ profile, and amino acid type. Finally, we compared the prediction results based on *Z*_SEQ_ with those of the methods using other structure-based features.

### 3.1. Distributions of SEQ for Catalytic and Noncatalytic Residues

**Figure 1:**
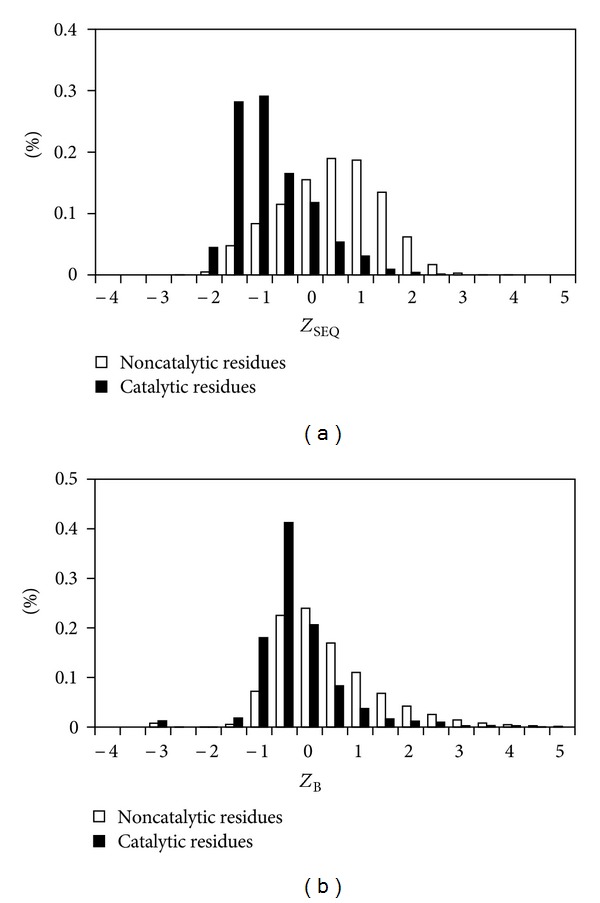
Distributions of (a) *Z*_SEQ_ and (b) *Z*_B_ profiles of catalytic and noncatalytic residues for the E760 dataset.

In this section, we compare the distributions of SEQ (sequence neighbor flexibility) for catalytic residues and noncatalytic residues.  Figure 1(a) displays the distributions of *Z*_SEQ_ when *n* = 1 (*n*: the number of flanking neighboring residues on sequence to calculate the average structural flexibility) for catalytic residues and noncatalytic residues for the E760 dataset. For comparison, the distributions of *Z*_B_ are also shown in Figure 1(b). The distributions of *Z*_SEQ_ for catalytic and noncatalytic residues show that catalytic residues are much less flexible and located in a more rigid context than noncatalytic residues. The phenomenon is much more significant using *Z*_SEQ_ than using *Z*_B_ as shown in Figure 1. There are 90% of catalytic residues having *Z*_SEQ_ ≤ 0 and 40% of noncatalytic residues having *Z*_SEQ_ ≤ 0. Only 81% of catalytic residues have *Z*_B_ ≤ 0 and 54% of noncatalytic residues have *Z*_B_ ≤ 0. SEQ and crystallographic B-factor are both based on the number and distances of neighbors around a given residue and are related to structural flexibility. The SEQ profile is a better and more reliable characteristic to identify catalytic residues than the B-factor profile. B-factor is easily affected by experimental conditions, crystal packing, existence of ligands, or temperature. Two structures of the same enzyme under different experimental conditions may have very different B-factor profiles but have almost identical crystal structures.

**Figure 2:**
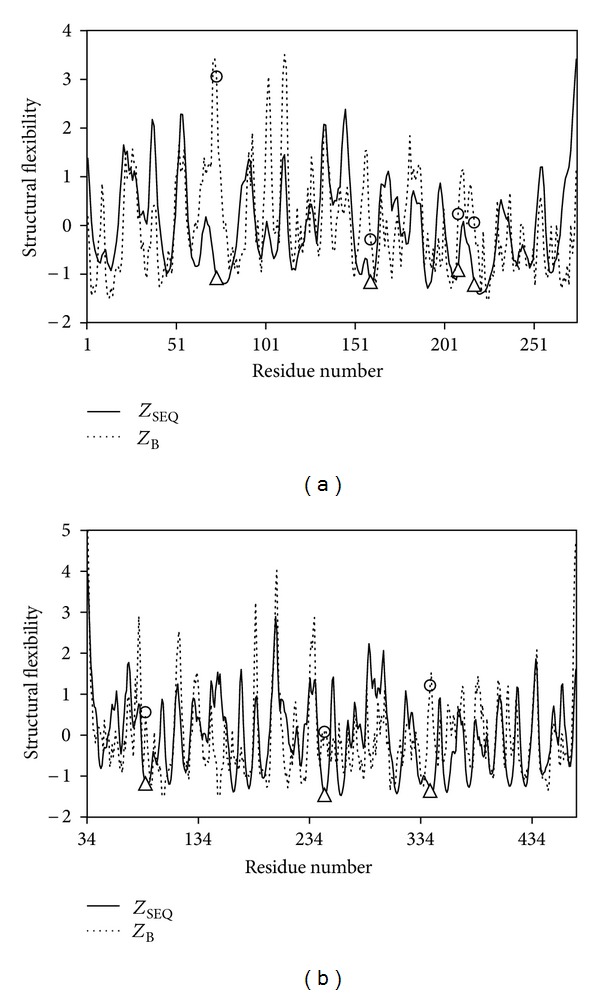
Comparison of SEQ and B-factor profiles for two example proteins. Comparison of *Z*_SEQ_ and *Z*_B_ profiles for (a) diaminopimelate epimerase (PDB: 1BWZ) and (b) levansucrase (PDB: 1OYG). The catalytic residues are labeled as triangles on *Z*_SEQ_ profile and as circles on *Z*_B_ profile. The catalytic residues of 1BWZ are Cys73, His159, Glu208, and Cys217. The catalytic residues of 1OYG are Asp86, Asp247, and Glu342.

Figure 2(a) shows the *Z*_SEQ_ of *n* = 1 (solid lines) and *Z*_B_ (dashed lines) profiles of two enzymes, diaminopimelate epimerase (PDB id: 1BWZ) and levansucrase (PDB id: 1OYG). The catalytic residues and noncatalytic residues are labeled as triangle and circle on *Z*_SEQ_ profiles and on *Z*_B_ profiles, respectively. It is obvious that the catalytic residues are located in the most structurally stable regions for the *Z*_SEQ_ profiles in both examples. The four catalytic residues (Cys73, His159, Glu208, and Cys217) of diaminopimelate epimerase are located near the centroid of the enzyme, forming a rigid catalytic spot. Cys73 and Cys217 are close to each other and connected by a disulfide bond. However, they have unusually high *Z*_B_ (3.06 and 0.06, resp.) but reasonable low *Z*_SEQ_ values (−1.08 and −1.22). His159 is partially buried by surrounding neighbors and has a quite low solvent accessible surface (SAS) of 3 Å^2^, calculated by the DSSP program [33]. It has a relatively low *Z*_B_ (−0.29) and an extremely low *Z*_SEQ_ (−1.16) comparing to other residues in the enzyme. Glu208 is relatively more exposed to solvent (SAS = 19 Å^2^) than His159. It has a high *Z*_B_ (0.23) but a very low *Z*_SEQ_ (−0.91). In the protein, the four catalytic residues are structurally rigid, having very low *Z*_SEQ_ and SAS values. However, their *Z*_B_ are high, especially for Cys73 that forms a disulfide bond with another catalytic residue, Cys217.

The catalytic site of the second example, levansucrase, is constituted of three catalytic residues, Asp86, Asp247, and Glu342, which are inside a cleft near the geometrical center of the protein. They are moderately accessible to solvent (with SAS: 20 Å^2^, 8 Å^2^, and 24 Å^2^, resp.) but are surrounded and thus strongly stabilized by a large number of residues because of their location. The *Z*_SEQ_ for these three catalytic residues, Asp86, Asp247, and Glu342, are −1.18, −1.46, and −1.36, respectively. Their *Z*_B_ values are surprisingly not low (0.56, 0.08, and 1.21, resp.). SEQ is a better estimation of structural flexibility than B-factor whether the location of residue is exposed to solvent or buried inside the protein.

### 3.2. Distributions of SEQ and STR Based on Different Parameter Settings

**Figure 3:**
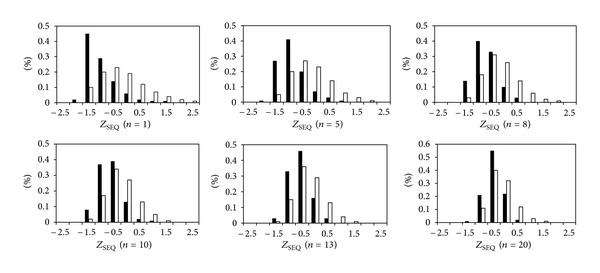
Distributions of *Z*_SEQ_ for catalytic and noncatalytic residues with different average window sizes.

In the previous section, we discussed the SEQ when parameter *n* = 1, that is, average WCN of target residue and its nearest two neighboring residues on sequence. Here, we extend the analysis to SEQ calculated based on different window sizes.  Figure 3 shows the distributions of SEQ for catalytic and noncatalytic residues with incremental *n* from 1 to 20. When *n* is set to 1, the SEQ distributions of catalytic and noncatalytic residues are obviously different. As window size increases, the differences between distributions decrease gradually. The difference between SEQ of catalytic and noncatalytic residues is less obvious when *n* is larger than 10.  Table 2 lists the prediction results when using SEQ with different *n*. The MCC obviously drops when *n* is equal or larger than 10. The results indicate that the sequence neighbors of catalytic residues are also structurally more rigid than noncatalytic residues in the range of *n* < 10.

**Figure 4:**
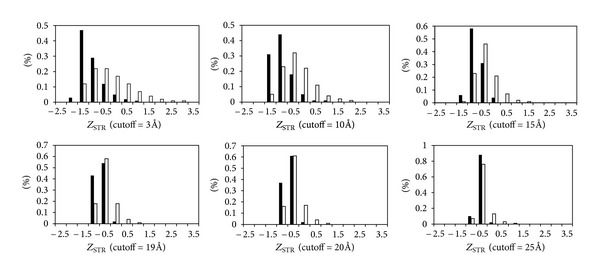
Distributions of *Z*_STR_ for catalytic and noncatalytic residues with different cut-off distances.

For STR, the cut-off distance for structurally neighboring residues is set from 3 Å to 25 Å as shown in Figure 4. Catalytic residues have structurally rigid neighbors for neighboring residues within 15 Å cut-off distance. When the cut-off distance is larger than 19 Å, catalytic and noncatalytic residues have similar STR distributions. The results suggest that catalytic residues are usually located in structurally stable environments and the surrounding neighboring residues within 15 Å are also relatively structurally rigid.

### 3.3. Prediction of Catalytic Residues Based on SEQ and STR

In this section, we discuss the prediction results using SVM with several different features, including amino acid type, SEQ (*n* = 1), STR (cutoff = 3 Å), B-factor profile, and their combinations. The prediction sensitivity, specificity, and MCC using different feature sets are listed in Table 1, including amino acid type (AA), SEQ and STR profiles, B-factor profile (B), combination of amino acid type, and SEQ (AA + SEQ).

The prediction results show that SEQ and STR are much better features than B-factor (MCC = 0.47 for SEQ and STR, 0.25 for B-factor) for identification of catalytic residues. The prediction performances of STR and SEQ are quite similar (sensitivity = 0.76 and 0.79, specificity = 0.70 and 0.69 for SEQ and STR, resp.). We selected SEQ for further-detailed analysis and comparison. Due to the fact that about 95% catalytic residues are polar or charged amino acids, prediction purely based on amino acid type have a MCC of 0.40, which is much higher than that of B-factor. However, the results also show that there are many false positives (specificity = 0.70), which means that catalytic residues have other unique features. SEQ provides information of structural flexibility of residues and their neighbors, which is complementary to amino acid type information. The prediction results that include SEQ and amino acid type show that catalytic residues can be more accurately identified using both features. The MCC is 0.51 and the sensitivity and specificity are 0.74 and 0.76, respectively. The prediction results based on combining SEQ (*n* = 1) and STR (cutoff = 3 Å) are also listed in Table 1. The prediction performance is similar to those based on SEQ or STR alone. The reason may be that the combination of SEQ and STR does not provide more information than SEQ or STR alone, thus not further improving the prediction results.

### 3.4. Comparison with Structure-Based Prediction Method

**Figure 5:**
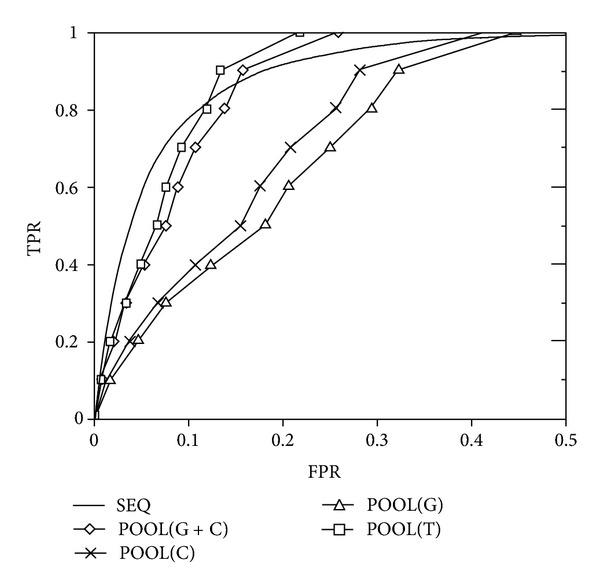
Comparison of ROC curves of SEQ and POOL using different features. The ROC curves are prediction results on a dataset of 160 enzymes. POOL features are denoted as POOL(T): residue electrostatic; POOL(G): structure geometry; POOL(C): sequence conservation; POOL(G + C): structure geometry combined with sequence conservation. The figure was remade from [6].

To test the performance of SEQ, we compared our prediction results with those of Partial Order Optimum Likelihood (POOL) [6], which combines residue electrostatic properties and structure geometry information to predict catalytic residues. POOL is one of the most successful structure-based prediction methods and it is able to work without needing sequence homology information. First, we directly compared the prediction results of SEQ and those of POOL on a dataset of 160 enzymes [6].  Figure 5 shows the ROC curves of SEQ and POOL based on different features including POOL(T): residue electrostatic properties, POOL(G): structure geometry feature, and POOL(C): sequence conservation. SEQ apparently outperforms POOL(C) and POOL(G) and performs better than POOL(T) and POOL(G + C) (POOL(G) combined with POOL(C)) when false-positive rate is smaller than 0.1. Under higher false-positive rates, POOL(T) and POOL(G + C) have better performance than that of SEQ. It is somewhat interesting that SEQ, which only uses structural rigidity, has comparable results as those of POOL, which uses residue biochemical features, evolutionary sequence conservation, and cleft shape. The results reported here are based on fivefold cross-validation on the dataset of 160 enzymes. The ratio between catalytic and noncatalytic residues is not changed (unbalanced) for each test fold.

**Figure 6:**
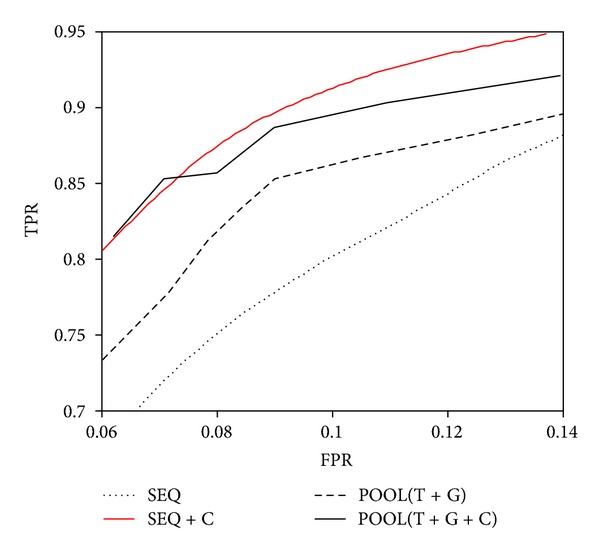
Comparison of ROC curves of SEQ and POOL on a dataset of 79 enzymes. Comparison of SEQ and POOL that combines all of its features, including POOL(T): residue electrostatic, POOL(G): structure geometry, dand POOL(C): sequence conservation. ROC curves of prediction using SEQ and SEQ combined with sequence conservation (SEQ + C) are both shown in the figure. The figure was remade from [6].

We also compared the results of SEQ and those of POOL(T + G) (residue electrostatic combined with structure geometry) on a dataset of 79 enzymes [24].  Figure 6 shows the ROC curves of SEQ (dotted line) and those of POOL. In the results of Figure 5, SEQ performs much better than POOL(G) and is comparable to POOL(T). When POOL(T) and POOL(G) are combined together (POOL(T + G)), it performs better than SEQ (Figure 6). POOL has the best performance when sequence conservation (POOL(C)) is further added (POOL(T + G + C)). To compare with their results, we combined SEQ and sequence conservation by PSI-BLAST. The results show that SEQ performs even better than POOL(T + G + C) when sequence conservation is added (thick solid line in Figure 6). It suggests that although SEQ can find out rigid regions in enzyme structures, amino acid information is still important for the identification of catalytic residues due to the fact that a large fraction of catalytic residues are polar amino acids. Using SEQ without any amino acid information to predict catalytic residue may result in some false positives, for example, rigid but nonpolar residues.

To further compare the results of SEQ and POOL, we have submitted two enzyme structures, diaminopimelate epimerase (PDB code: 1BWZ) and levansucrase (PDB code: 1OYG), to the POOL webserver. For diaminopimelate epimerase, the four catalytic residues, Cys73, His159, Glu208, and Cys217, are ranked 18, 3, 6, and 59, respectively, by POOL (residues higher ranked in POOL are more probable to be catalytic residue). In Figure 2(a), it is clear that SEQ correctly identifies all catalytic residues, which are located on the globally most rigid (small SEQ values) regions. For levansucrase, the three catalytic residues, Asp86, Asp247, and Glu342, are ranked 6, 5, and 2, respectively, by POOL. In Figure 2(b), the three catalytic residues locate in the most structurally rigid regions according to SEQ. It is also interesting that residues Glu340, Glu262, and Tyr411 are ranked 1, 3, 4, respectively, by POOL. These residues are located in relatively rigid regions in the SEQ profile.  Table 3 lists the prediction rank of POOL and our prediction using SEQ (*n* = 1) for each catalytic residue in several example proteins. The rank of our prediction is based on the probability of a residue predicted to be catalytic residue based on the function provided by the LIBSVM software. The results show that our prediction results are in general better than or comparable to those of POOL in these examples.

### 3.5. Discussions on Related Prediction Methods

Here we discuss related catalytic residue prediction methods, including their features, datasets, and prediction performance. Petrova and Wu [34] used 24 features, including sequence-based features: amino acid type, sequence conservation, and structure-based and chemical features: shape of local structure, solvent accessible surface, structural flexibility, and hydrogen bonding. A dataset of 79 enzymes containing totally 23,664 residues and 254 catalytic residues was used for performance evaluation. Among these features, the seven best features were selected. The MCC of using different combinations of these features ranges from 0.52 to 0.74 and the sensitivities range from 0.88 to 0.89. To avoid the problems in SVM training due to the extremely unbalanced number of catalytic and noncatalytic residues, they used a similar strategy we used here for SVM training and predicting. The strategy is to build a subset that includes all catalytic residues and equal number of noncatalytic residues selected randomly. It is interesting to note that, without using the sequence conservation feature, the prediction MCC is only 0.52.

A more recent study by Cilia and Passerini [35] models spherical regions around target residues and extracts the properties of their content such as physicochemical properties, atomic density, flexibility, and presence of water molecules. They performed the prediction using SVM with these structural features and other sequence-based features: amino acid type and sequence conservation. 

Amitai et al. [11] applied graph theory to catalytic residue prediction by converting protein structure to network in which the graph nodes are residues and graph edges are residues interactions. They found that catalytic sites have higher network closeness than noncatalytic residues. The features used in their prediction included the closeness feature, solvent accessible surface, and sequence conservation using a dataset of 178 enzymes. 

Wie et al. [12] used a computational method called Theoretical Microscopic Titration Curves (THEMATICS), which computes theoretical electrostatic properties of residues based on structure information. They simply set a threshold to identify catalytic residues, that is, each residue was assigned a score calculated by THEMACTICS and residues having score greater than the threshold are predicted as catalytic residues. The dataset used contains 169 enzymes, including 594 annotated catalytic residues. The sensitivities using different thresholds range from 0.41 to 0.63. THEMATICS is then combined with other structure feature and is called POOL, with which we chose to compare our results. The reason we compared with POOL is that POOL is the most accurate structure-based prediction method. There are other methods having better prediction results combining complicated sequence features and structure features [35]. However, they usually do not provide prediction results only using structure features.

## 4. Conclusions

In this work, we calculated theoretical structural flexibility for catalytic residues and their sequence or structure neighboring residues. We found that catalytic residues are in general located in structurally less flexible context. We show that the theoretical structure flexibility (SEQ and STR) we used is better than B-factor for identification of catalytic residues. For a dataset of 760 enzymes of low pairwise sequence identity, the difference of SEQ distributions between catalytic and noncatalytic residues are more obvious than that of B-factor. The prediction results of SEQ are much better then those of B-factor. The MCC, sensitivity, and specificity of prediction are 0.74, 0.76, and 0.51, respectively, using SEQ combined with amino acid type information. The prediction results using SEQ are comparable to or better than those of other structure-based features. Most current prediction methods need homology information, for example, sequence conservation from PSI-Blast, and require the existence of sequence or structure similar proteins. SEQ and STR are calculated from single-protein structure and do not require any homology information. They may be further applied to the detection of enzyme function-related sites, like protein ligand binding site, metal binding site, or protein-protein interaction hotspot residues.

## Figures and Tables

**Table 1:** Prediction performances using SVM with different features.

Feature set	Performance		
	Sensitivity	Specificity	MCC
AA^a^	0.70	0.70	0.40
SEQ^b^	0.76	0.70	0.47
STR^c^	0.79	0.69	0.47
SEQ + STR^d^	0.78	0.66	0.45
B-factor	0.63	0.62	0.25
AA + SEQ	0.74	0.76	0.51

^a^Amino acid type.

^b^SEQ with *n* = 1.

^c^STR with cut-off distance = 3 Å.

^d^SEQ with *n* = 1 combined with STR with cut-off distance (3 Å).

**Table 2:** Prediction results when using SEQ with different *n* parameters.

*n *	Performance		
	Sensitivity	Specificity	MCC
1	0.76	0.70	0.47
5	0.75	0.71	0.47
8	0.79	0.65	0.44
10	0.80	0.57	0.36
13	0.78	0.55	0.36
20	0.77	0.41	0.36

**Table 3:** Comparison of rank of catalytic residues for predictions using SEQ and POOL.

PDB ID and chain	Catalytic residue	Rank	
		SEQ (*n* = 1)^a^	POOL
1BWZ:A	C73	8	18
	H159	10	3
	E208	5	6
	C217	7	59
1OYG:A	D86	8	6
	D247	9	5
	E342	7	2
1A95:C	D88	1	1
	D89	2	2
	D92	7	8
	K115	6	27
1EC9:A	K205	3	10
	K207	2	4
	D313	8	3
	H339	7	1
	D366	5	12
1EHK:A	H233	2	6
	Y237	6	5
	H384	3	8
	F385	4	66
	H386	5	26
	R449	1	4
	R450	7	7

^a^The rank of prediction using SEQ is based on the probability of a residue predicted to be catalytic residue.
